# Supplementary material for: Empirical methods for controlling false positives and estimating confidence in ChIP-Seq peaks
Source: BMC Bioinformatics. 2008 Dec 5;9:523. doi: 10.1186/1471-2105-9-523 (PMC2628906; doi:10.1186/1471-2105-9-523)
Supplement: Additional File 1 — A variety of html documents from the USeq web site detailing the available applications, their best usage, output file type descriptions, command line menus, etc. [file 1471-2105-9-523-S1.zip › USeqUserGuides/JavaDoc.zip!/JavaDoc/util/bio/parsers/gff/Gff3Parser.html]

Gff3Parser


|  |  |  |
| --- | --- | --- |
| **Overview** \| **Package** \| **Class** \| **Use** \| **Tree** \| **Deprecated** \| **Index** \| **Help** | |  |
| **PREV CLASS**   **NEXT CLASS** | **FRAMES**    **NO FRAMES**     **All Classes** |  |
| SUMMARY: NESTED \| FIELD \| CONSTR \| METHOD | DETAIL: FIELD \| CONSTR \| METHOD |  |


---


## util.bio.parsers.gff Class Gff3Parser

```
java.lang.Object
  util.bio.parsers.gff.Gff3Parser
```

---

``` public class Gff3Parser extends java.lang.Object ```

For parsing a GFF3 text file into GFF3Feature objects, be sure to set the regExTypes String filter to match which types you want to save!

---

**Constructor Summary**

`Gff3Parser()`

`Gff3Parser(java.io.File GFF3File)`


| **Method Summary** | |
| --- | --- |
| `Gff3Feature[][]` | `getChromSplitFeatures()`             Returns the features split by chromosome. |
| `Gff3Feature[]` | `getFeatures()` |
| `java.io.File` | `getGFFFile()` |
| `java.lang.String` | `getRegExTypes()` |
| `static void` | `main(java.lang.String[] args)` |
| `boolean` | `parseIt(java.io.File GFF3File)` |
| `boolean` | `printNotes()` |
| `void` | `setGFFFile(java.io.File file)` |
| `void` | `setPrintNotes(boolean printNotes)` |
| `void` | `setRegExTypes(java.lang.String regExTypes)`             Use something like -> gene\|ncRNA\|snoRNA\|tRNA\|rRNA\|transposable\_element\|pseudogene\|CDS\|mRNA\|exon Only those gff lines with a type matching this regular expression will be made into GFF3Feature's. |
| `void` | `setRelax(boolean relax)` |
| `void` | `subtractOneFromFeatures()`             Subract one from start and end of each feature. |

**Methods inherited from class java.lang.Object**

`equals, getClass, hashCode, notify, notifyAll, toString, wait, wait, wait`

**Constructor Detail**

### Gff3Parser

```
public Gff3Parser(java.io.File GFF3File)
```

---


### Gff3Parser

```
public Gff3Parser()
```


**Method Detail**

### parseIt

```
public boolean parseIt(java.io.File GFF3File)
```

---


### subtractOneFromFeatures

```
public void subtractOneFromFeatures()
```

:   Subract one from start and end of each feature. Use for converting to zero base coordinates.

---


### getChromSplitFeatures

```
public Gff3Feature[][] getChromSplitFeatures()
```

:   Returns the features split by chromosome.

---


### main

```
public static void main(java.lang.String[] args)
```

---


### getFeatures

```
public Gff3Feature[] getFeatures()
```

---


### getGFFFile

```
public java.io.File getGFFFile()
```

---


### setGFFFile

```
public void setGFFFile(java.io.File file)
```

---


### printNotes

```
public boolean printNotes()
```

---


### setPrintNotes

```
public void setPrintNotes(boolean printNotes)
```

---


### getRegExTypes

```
public java.lang.String getRegExTypes()
```

---


### setRegExTypes

```
public void setRegExTypes(java.lang.String regExTypes)
```

:   Use something like -> gene|ncRNA|snoRNA|tRNA|rRNA|transposable\_element|pseudogene|CDS|mRNA|exon
    Only those gff lines with a type matching this regular expression will be made into GFF3Feature's.
    If you want everything set this to '.+' .
    If you don't know what types are present in the gff file, run this parser and it will print all of the types.

---


### setRelax

```
public void setRelax(boolean relax)
```


---


|  |  |  |
| --- | --- | --- |
| **Overview** \| **Package** \| **Class** \| **Use** \| **Tree** \| **Deprecated** \| **Index** \| **Help** | |  |
| **PREV CLASS**   **NEXT CLASS** | **FRAMES**    **NO FRAMES**     **All Classes** |  |
| SUMMARY: NESTED \| FIELD \| CONSTR \| METHOD | DETAIL: FIELD \| CONSTR \| METHOD |  |


---
